# Loss of Neutrophils and SLPI Reshapes Early Mucosal Inflammatory Responses During Intranasal Vaccination

**DOI:** 10.1002/eji.70282

**Published:** 2026-09-16

**Authors:** Oktawia Osiecka, Mariia Tyshchenko, Ivan Sinkevich, Natalia Pocałuń, Joanna Cichy, Jakub M. Kwiecinski, Ewa Oleszycka, Mieszko M. Wilk

**Affiliations:** ^1^ Department of Immunology Faculty of Biochemistry, Biophysics, and Biotechnology Jagiellonian University Kraków Poland; ^2^ Doctoral School of Exact and Natural Sciences Jagiellonian University Kraków Poland; ^3^ Department of Microbiology Faculty of Biochemistry, Biophysics, and Biotechnology Jagiellonian University Kraków Poland

**Keywords:** coagulation, metalloproteinase, nasal vaccine, neutrophils, protease inhibitors

## Abstract

Vaccines delivered via the nasal route have attracted increasing attention for their capacity to engage the mucosal immune system at the primary site of pathogen entry. However, the early innate mechanisms that shape local immune responses following nasal immunisation remain poorly defined. Here, we identified previously uncharacterised tissue‐derived mediators induced during vaccination. Using an intranasally administered whole‐cell pertussis (wP) vaccine that recapitulates key features of *Bordetella pertussis* infection and elicits long‐lasting immunity, we show that nasal delivery triggers neutrophil recruitment along with a marked upregulation of secretory leukocyte protease inhibitor (SLPI). Employing SLPI‐ and neutrophil‐deficient mouse models, we demonstrate that SLPI and neutrophils influence the production of key mediators associated with coagulation (PAI‐1) and tissue remodelling (MMP‐3 and MMP‐9) within the airway. Notably, neutrophils also modulate early local total IgA release. Together, these findings suggest a coordinated and context‐dependent interplay between cellular and soluble innate components that shape early mucosal immune responses. Furthermore, identified mediators may represent candidate biomarkers in the development of intranasal vaccines against *B. pertussis* and other pathogens.

## Graphical abstract

Intranasal vaccination induces pulmonary neutrophil accumulation and SLPI release, accompanied by changes in soluble mediators associated with tissue remodelling, coagulation, and total IgA.

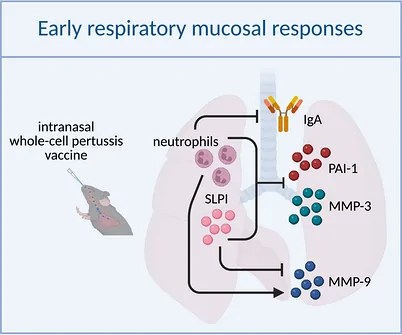

## 1 Introduction

The development of effective vaccination strategies remains a cornerstone of public health, with increasing interest in distinguishing systemic and mucosal immunity [1, 2]. The most common route of vaccine administration is intramuscular injection, which primarily induces systemic immunity and reduces the severity of the disease. However, intramuscular vaccines have limited impact on preventing pathogen entry and transmission at mucosal sites. To address this, alternative delivery methods, such as oral and intranasal vaccination, have been developed [2]. For respiratory pathogens, several intranasal vaccines are already in clinical use, for example against influenza and COVID‐19 [3, 4]. Other respiratory diseases could also benefit from intranasal vaccine administration, including whooping cough (pertussis), caused by *Bordetella pertussis* (Bp) [5, 6]. Bp infects the respiratory tract and can cause severe illness in infants and young children, while also posing a significant risk to the elderly and individuals with comorbidities [7]. Due to the severity of pertussis, intramuscular vaccines were developed in the 20th century. The widespread introduction of whole‐cell pertussis vaccines (wP) in the 1940s–1950s led to a substantial reduction in the incidence of disease in many countries. In contrast, the introduction of acellular pertussis vaccines (aP) in the late 1990s has been associated with a resurgence of whooping cough in countries that transitioned from wP to aP vaccines [5]. This has been attributed to the aP vaccines, which induce shorter‐lived immunity compared with the wP vaccines [8]. The failure to eliminate pertussis outbreaks despite widely available vaccines indicates that novel strategies are needed to enhance herd immunity. In addition, novel reliable biomarkers are required that could identify a protective immune vaccine response. Recent studies have shown that tissue‐resident memory cells (Trm) and secretory IgA are required for protective immunity against pertussis following natural infection and nasal vaccination with a live attenuated pertussis vaccine [9, 10]. However, the early regulation of local immune responses in the respiratory mucosa, particularly after intranasal vaccine administration, remains poorly understood. Thus, focusing on soluble factors and their cellular sources can enhance the understanding of the local protective immunity induced by vaccination.

Secretory leukocyte protease inhibitor (SLPI) is a multifunctional molecule that acts as a protease inhibitor, antimicrobial peptide, and modulator of local inflammation, preventing tissue damage [11]. SLPI is constitutively expressed in mucosal tissues, but its local role in the respiratory system is only beginning to be appreciated. For instance, SLPI has been shown to control local allergic responses, protecting against severe IFN‐γ–mediated lung inflammation during allergic asthma [12]. In addition, SLPI expression is affected by local respiratory microbiota, such as *Bordetella pseudohinzii*, which can colonize mouse airways and protect against allergic airway inflammation [13]. Furthermore, a key cytokine driving mucosal immunity, IL‐17, regulates SLPI expression [13]. As IL‐17 is critical to induce protective immunity against nasal infection with Bp by mobilizing neutrophils and driving secretory IgA [10], SLPI might be a key player in driving vaccine‐induced mucosal protective immunity.

In this project, we specifically aimed to elucidate the contribution of SLPI and SLPI^+^ cells to local tissue remodelling and early immune responses after intranasal vaccination. We investigated the response to intranasal whole‐cell pertussis vaccination, which mimics natural infection and induces long‐lasting immunity.

## 2 Materials and methods

### 2.1 Reagents and Antibodies

The following reagents were used: acetic acid (Poch S.A.); BSA (BioShop); Brilliant Blue R‐250 (Sigma‐Aldrich); calcium chloride dihydrate (Sigma‐Aldrich); collagenase‐D (Roche Diagnostics); DNase‐I (Sigma‐Aldrich); Foxp3/transcription factor fixation/permeabilisation concentrate and diluent (Thermo Fisher Scientific); Gelatin type A from porcine skin (Sigma‐Aldrich); Heat‐inactivated fetal bovine serum (Thermo Fisher Scientific); intracellular fixation buffer (Thermo Fisher Scientific); 2× Laemmli sample buffer (Bio‐Rad); methanol (Chemland); PBS (PAN‐Biotech); penicillin‐streptomycin (Thermo Fisher Scientific); permeabilisation buffer (10×) (Thermo Fisher Scientific); 1× RBC lysis buffer (Thermo Fisher Scientific); RPMI 1640 (Biowest); Whole‐cell pertussis vaccine (DTP; diphtheria‐tetanus‐pertussis vaccine, IBSS BIOMED S.A., Poland) referred as “whole‐cell pertussis vaccine” or “wP vaccine”; streptavidin PE (BD); Tris (BioShop); TMB substrate reagent set (BD); Tween‐20 (Sigma‐Aldrich); Triton X‐100 (Poch S.A.); WesternBright ECL HRP substrate (Advansta); Zinc chloride (Sigma‐Aldrich), Zombie Aqua Fixable Viability Kit (Biolegend). The following antibodies were used: anti‐MHC II Alexa Fluor 700 (0.02 µg/100 µL; clone M5/114.15.2, Thermo Fisher Scientific), anti‐Ly6C PE‐Cy7 (0.005 µg/mL; clone HK1.4, Biolegend), anti‐B220 BV786 (0.2 µg/100 µL; clone RA3‐6B2, BD), anti‐CD11b BV650 (0.04 µg/100 µL; clone M1/70; BD); anti‐CD11c BV421 (0.2 µg/100 µL; clone HL3, BD), anti‐CD11c PerCPCy5.5 (0.2 µg/100 µL; clone N418; Thermo Fisher Scientific), anti‐Ly6G BV711 (0.2 µg/100 µL; clone 1A8; BD); anti‐SiglecF PE (0.1 µg/100 µL; clone E50‐2440; BD), anti‐SiglecF PerCPCy5.5 (0.1 µg/100 µL; clone E50‐2440; BD); anti‐CD45 APC‐eFluor 780 (0.2 µg/100 µL; clone 104; Thermo Fisher Scientific); anti‐CD16/CD32 monoclonal antibodies (0.25 µg/100 µL; clone 93; Thermo Fisher Scientific); anti‐mouse SLPI‐biotin (0.067 µg/100 µL; R&D Systems); InVivoMAb anti‐mouse/rat IL‐17A (Biozol); InVivoMAb mouse IgG1 isotype control (Biozol).

### 2.2 Mice

*Slpi* ^−/−^ mice were received from Dr Sharon M. Wahl [14] and were used with corresponding C57BL/6 (referred to as WT) mice. Transgenic mice *Mcl1* ^WT^ and *Mcl1* ^MKO^ were established from colonies of C57BL/6 *Lyz2* ^Cre/Cre^ and *Mcl1* ^flox/flox^ mice received from Attila Mócsai (Semmelweis University School of Medicine, Hungary) and were maintained by breeding of heterozygotes *Lyz2* ^Cre/Cre^ *Mcl1* ^flox/+^ [15]. Mice were housed in a pathogen‐free animal facility at the Faculty of Biochemistry, Biophysics and Biotechnology of Jagiellonian University. The mice used in the experiments were matched in sex and age, and were 8–12 weeks old. All animal procedures and experiments were approved by the first Local Institutional Animal Care and Use Committee (IACUC) in Krakow (approval numbers 76/2023 and 956/2024).

### 2.3 *In Vivo* Models

For intraperitoneal (i.p.) vaccination, mice were injected with 1/20 human dose of a wP vaccine diluted in PBS (total 200 µL volume). PBS was used as a control. For intranasal (i.n.) vaccination, mice were anaesthetised with 2% isoflurane and then administered with 1/20 human dose of a wP vaccine diluted in PBS (total 40 µL volume, 20 µL per nostril). PBS was used as a control. For IL‐17A neutralisation, mice were injected i.p. with anti‐IL‐17Ab at 200 µg/mouse 6 h before i.n. immunisation with the wP vaccine and every 2 days (on days 2, 4 and 6) after vaccination. A corresponding isotype control was used for the control mice. Mice were sacrificed at the time indicated in the figure legends.

### 2.4 Serum Isolation

Blood was collected from the mice's eyes and, after approximately 1 h at RT, centrifuged (500*g*, 20 min, RT). Obtained serum was centrifuged (3000*g*, 15 min, RT) and frozen for further analysis.

### 2.5 Isolation of BAL Fluid and Lung Cells

BAL fluid was harvested from mice after gentle injection and aspiration of 0.5 mL PBS (for proteome) or 1 mL PBS using a 1 mL syringe and a catheter inserted into the mouse trachea. The lavage fluids were centrifuged (400*g*, 5 min, 4°C), and supernatants and cells were collected for further analysis. Lungs were extracted and chopped. Samples were digested with collagenase‐D (1 mg/mL) and DNase I (10 µg/mL) in a volume of 1 mL for 1 h at 37°C with agitation. Lungs were passed through a 70 µm cell strainer (pluriSelect) to obtain a single‐cell suspension. 1X RBC Lysis Buffer was used, followed by washing with PBS.

### 2.6 Flow Cytometry

Cells were incubated with LIVE/DEAD Aqua and then blocked with anti‐CD16/CD32 antibodies, followed by staining with directly conjugated anti‐mouse antibodies. Cells were fixed with IC Fixation Buffer in PBS (1:1). For detection of intracellular SLPI, cells were fixed, permeabilised, and stained with Foxp3/transcription factor fixation/permeabilisation according to the manufacturer's instructions. Cells were incubated with primary biotinylated antibody followed by PE−streptavidin. Counting beads (Thermo Fisher Scientific) were used to obtain absolute counts of cells. UltraComp eBeads (Thermo Fisher Scientific) were used for compensation set‐up. Fluorescence minus one (FMO) samples were used as controls. Flow cytometry was performed on LSRII (BD Biosciences) and the data were analysed using the software FACSDiva (BD Biosciences) and FlowJo version 10.10.0 (BD). The gating strategies are provided in Figure S1.

### 2.7 ELISA

Concentrations of mouse SLPI (Cat. No. DY1735‐05), MMP‐9 (Cat. No. DY6718), MMP‐3 (Cat. No. DY548), serpin E1/PAI‐1 (Cat. No. DY3828‐05), and neutrophil elastase (Cat. No. DY4517‐05) were measured using kits from R&D Systems and concentration of IgA antibodies were measured using Mouse IgA Uncoated ELISA Kit (Cat. No. 88‐50450‐22, Invitrogen, Thermo Fisher Scientific) according to the manufacturer's instructions.

### 2.8 Antibody‐specific Responses

Heat‐killed Bp was prepared as previously described [16]. *B. pertussis*‐specific IgA was quantified in BAL fluid by ELISA using plate‐bound HKBp (10^7^ CFU/mL) and HRP‐conjugated goat anti‐mouse IgA (1:1000, Southern Biotech). The reaction was developed using BD OptEIA TMB substrate reagent set (Waters Biosciences) and 0.1 M H_2_SO_4_, and absorbance was detected at 450 nm.

### 2.9 Proteome Profiler Array

Samples were prepared by pooling 100 µL BAL fluids per mouse (400 µL total per membrane) or by pooling 6.25 µL serum per mouse (25 µL total per membrane). Proteome Profiler mouse angiogenesis array kit (Cat. no. ARY015, R&D Systems, Bio‐Techne) was used according to the manufacturer's instructions.

### 2.10 Zymography

BAL fluid samples were diluted in non‐reducing loading dye. Samples were incubated at 37°C for 30 min and resolved by SDS‐PAGE on 7% acrylamide gels containing 0.67 mg/ml gelatin. After electrophoresis, gels were washed twice for 30 min in wash buffer (2.5% Triton X‐100 in 50 mM Tris, pH 7.7) and incubated in activation buffer (50 mM Tris, pH 7.7, 5 mM CaCl_2_, 1 µM ZnCl_2_) for 16 h at 37°C. Gels were then stained with 0.5% Brilliant Blue R in 40% methanol and 10% acetic acid and visualised by ChemiDoc.

### 2.11 Analysis of Publicly Available Expression Data

Raw counts for scRNA‐seq analysis of lung cells were obtained from GSE310116 [17]. Briefly, low‐quality cells were filtered out using thresholds such as number of genes per cell, number of reads per cell and percentage of mitochondrial genes per cell. Doublets were removed using scDblFinder. Data integration and clustering were performed using Harmony in Seurat with default parameters and 21 principal components. Clusters were merged manually to show cell populations with unique markers.

### 2.12 Statistical Analysis

The statistics were analysed using GraphPad Prism 10 (GraphPad Software). Each number stated in the figure legend represents an individual mouse. The pooled experiments present all individual mice with the total number stated in the legends of the figures. The number of pooled experiments is stated in the figure legends. Data were presented as mean (±SEM). The unpaired two‐tailed *t*‐test was used when only two groups were compared. Multiple groups of a single mouse genotype were compared using one‐way ANOVA with multiple comparisons and Tukey post hoc test. Multiple groups with two mouse genotypes were compared using two‐way ANOVA with multiple comparisons and Tukey post hoc test.

## 3 Results

### 3.1 wP Vaccine Induces Local SLPI Release

**FIGURE 1:**
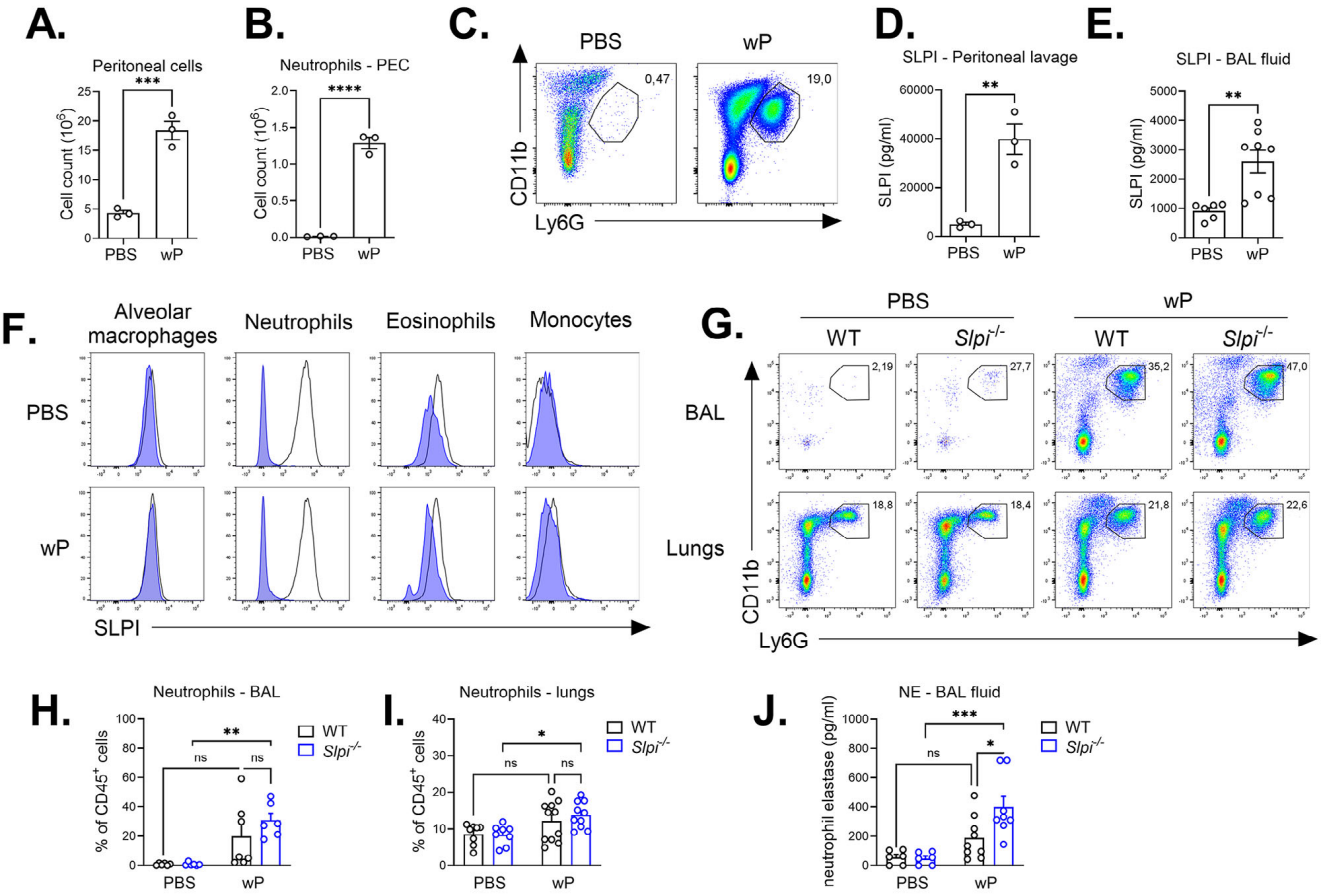
Intranasal wP vaccine induces local SLPI release and SLPI^+^ neutrophil accumulation. (A–D) WT mice were administered with PBS (as a control) or wP vaccine intraperitoneally on day 0 and day 28. After 14 days, peritoneal cells and fluid were isolated. (A) Total cell number and (B) neutrophils were analysed by flow cytometry. (C) Representative flow cytometry analysis of neutrophils in the peritoneal cavity as in (B). (D) Levels of SLPI in the peritoneal cavity were analysed by ELISA. (E) WT mice were administered with PBS or wP vaccine intranasally. On day 7, BAL fluid was isolated and analysed for SLPI levels by ELISA. (F–J) WT and *Slpi^−/−^* mice were administered with PBS or wP vaccine intranasally on day 0. After 7 days, lungs, BAL cells and fluid were isolated. (F) Representative SLPI expression in alveolar macrophages, neutrophils, eosinophils and monocytes from lungs of WT (black line) and *Slpi* ^−/−^ mice (shaded histogram). (G–I) Neutrophil frequency in (H) BAL and (I) lungs was analysed by flow cytometry. (G) Representative flow cytometry staining of neutrophils (CD11b^+^Ly6G^high^ cells) in BAL and lungs. (J) Levels of NE were analysed by ELISA. (A, B, D) Each point represents one mouse. Data represent 3 mice per experimental group from one experiment. Error bars show means ± SEM. **p < 0.01, ***p < 0.001, ****p < 0.0001 by unpaired two‐tailed *t*‐test. (E) Each point represents one mouse. Data represent 6–8 mice per experimental group pooled from two independent experiments. Error bars show means ± SEM. **p < 0.01 by unpaired two‐tailed *t*‐test. (H–J) Each point represents one mouse. Data represent 6 to 11 mice per experimental group pooled from at least two independent experiments. Error bars show means ± SEM. *p < 0.05, **p < 0.01, ***p < 0.001 by two‐way ANOVA, Tukey post hoc test.

The well‐established mouse intraperitoneal (i.p.) vaccination model induces an early innate activation followed by adaptive immunity [9, 18]. However, the impact of innate immune cells on the quality and durability of humoral and cellular adaptive responses is only beginning to be understood. To examine whether pertussis vaccination induces sustained local changes in innate cell populations, mice were immunised twice with wP vaccine and the local response was analysed 14 days post‐final immunisation. wP vaccine led to a significant increase in total peritoneal cell numbers compared with PBS (Figure 1A). In particular, neutrophils were accumulating in the peritoneal cavity (Figure 1B,C). Concurrently, there was also a significant upregulation of SLPI after wP vaccination in peritoneal lavage fluid (Figure 1D).

To assess whether a similar mechanism can be observed after intranasal (i.n.) vaccination, mice received a single dose of wP vaccine and innate responses were evaluated 7 days later. Consistent with the i.p. model, i.n. vaccination induced local SLPI upregulation (Figure 1E). To investigate whether SLPI played a role in the recruitment of innate immune cells, the response to wP was compared in wild‐type (WT) and *Slpi^−/−^* mice. Although neutrophils were identified as the primary producers of SLPI (Figure 1F), their accumulation in bronchoalveolar lavage fluid (BAL) and lung tissue did not differ significantly between WT and *Slpi^−/−^* mice following wP vaccination at day 7 (Figure 1G–I). However, when neutrophil elastase (NE) levels were analysed in BAL fluid, NE was significantly upregulated in SLPI‐deficient mice immunised with wP (Figure 1J). These findings suggest that local induction of SLPI is not essential for cell recruitment in response to wP vaccination but might impact neutrophil functionality and local release of immune modulators.

### 3.2 SLPI Inhibits Excessive Immune Responses in the Lungs

**FIGURE 2:**
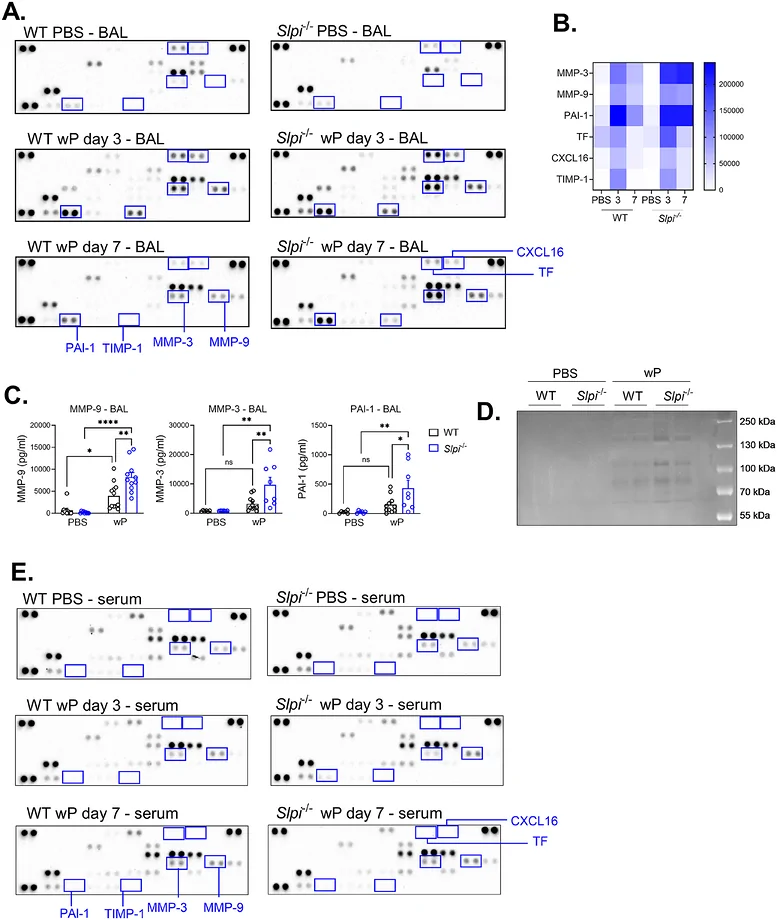
SLPI regulates the secretion of selected mediators involved in tissue remodelling and the coagulation cascade. (A–E) WT and *Slpi^−/−^* mice were administered PBS or wP intranasally. After 3 or 7 days, BAL was isolated and analysed. (A) Proteome array analysis of pooled BAL fluids of 4 mice per group. (B) Heatmap of densitometric analysis of membranes from (A). (C) Analysis of MMP‐9, MMP‐3, and PAI‐1 levels in PBS and wP samples from day 7 post‐administration. (D) Representative zymography of BAL fluid samples at day 7. Each lane represents one mouse. (E) Proteome array analysis of pooled serum of 4 mice per group. (A, E) Data represent one measurement per membrane from samples pooled from 4 mice. (C) Each point represents one mouse. Data from 6 to 12 mice per experimental group were pooled from at least two independent experiments. Error bars show means ± SEM. *p < 0.05, **p < 0.01, ****p < 0.0001 by two‐way ANOVA, Tukey post hoc test.

Next, we evaluated whether the absence of SLPI affects soluble mediators released in the lungs during vaccination. Proteome array membranes capable of detecting more than 50 factors associated with angiogenesis and tissue remodelling were used (Figure 2A) as SLPI has previously been shown to modulate MMP‐9 [19, 20]. Analysis of pooled BAL fluid of WT mice revealed a transient increase in mediators including matrix metalloproteinase‐3 (MMP‐3), matrix metalloproteinase‐9 (MMP‐9), plasminogen activator inhibitor‐1 (PAI‐1), tissue factor (TF), TIMP‐1, and CXCL16 at day 3 post‐vaccination. The levels of these mediators declined by day 7, indicating a temporal response. In contrast, while SLPI‐deficient mice upregulated the same mediators at day 3 as WT mice, there was a difference in response at day 7. TF, TIMP‐1 and CXCL16 decreased in SLPI‐deficient mice, while MMP‐3, MMP‐9 and PAI‐1 were upregulated when compared with WT mice (Figure 2A,B). As proteome profiler analysis was performed on pooled samples, these data are considered exploratory. Therefore, the levels of MMP‐3, MMP‐9 and PAI‐1 in BAL were further validated by ELISA (Figure 2C). Furthermore, BAL fluid from wP‐immunised *Slpi^−/−^* mice has higher MMP‐9 activity than from WT mice (Figure 2D). Notably, no changes were observed in circulating mediators in pooled samples of sera (Figure 2E), suggesting that the intranasal vaccine specifically regulates local lung responses. Collectively, these results suggest that SLPI modulates the temporal dynamics of soluble mediators in the respiratory mucosa after intranasal vaccination.

### 3.3 Neutrophils Regulate Local Immune Responses in the Lungs During Vaccination

**FIGURE 3:**
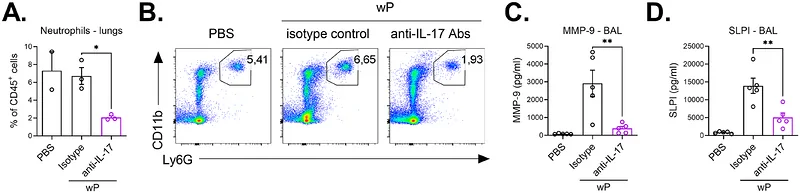
Effect of IL‐17 on innate response induced by wP vaccine. (A–C) WT mice were injected intraperitoneally with control or IL‐17‐blocking antibodies, followed by intranasal administration of wP vaccine. Control mice received PBS only. After 7 days, BAL and lungs were isolated. (A, B) The presence of neutrophils in the lungs was examined by flow cytometry analysis. Analysis of MMP‐9 (C) and SLPI (D) levels in BAL by ELISA. (A) Each point represents one mouse. Data represent 2–3 mice per experimental group from one experiment. Error bars show means ± SEM. *p < 0.05 by one‐way ANOVA, Tukey post hoc test. (C, D) Each point represents one mouse. Data represent 5 mice per experimental group pooled from two independent experiments. Error bars show means ± SEM. **p < 0.01 by one‐way ANOVA, Tukey post hoc test.

IL‐17 has been shown to regulate SLPI expression and neutrophil recruitment to the lungs in mice infected with *Bordetella pseudohinzii* [13]. Thus, we wanted to determine whether IL‐17 regulates SLPI‐driven responses in our model. Mice were treated with anti‐IL‐17 blocking antibodies during intranasal wP immunisation. IL‐17 blockade resulted in a significant reduction in neutrophil infiltration into the lungs (Figure 3A,B). Consistent with the role of neutrophils as the main source of MMP‐9, its levels were also reduced in vaccinated mice receiving IL‐17 blockade (Figure 3C). Additionally, IL‐17 blockade led to a decrease in SLPI secretion (Figure 3D), indicating that IL‐17 signalling contributes to both SLPI production and neutrophil infiltration.

**FIGURE 4:**
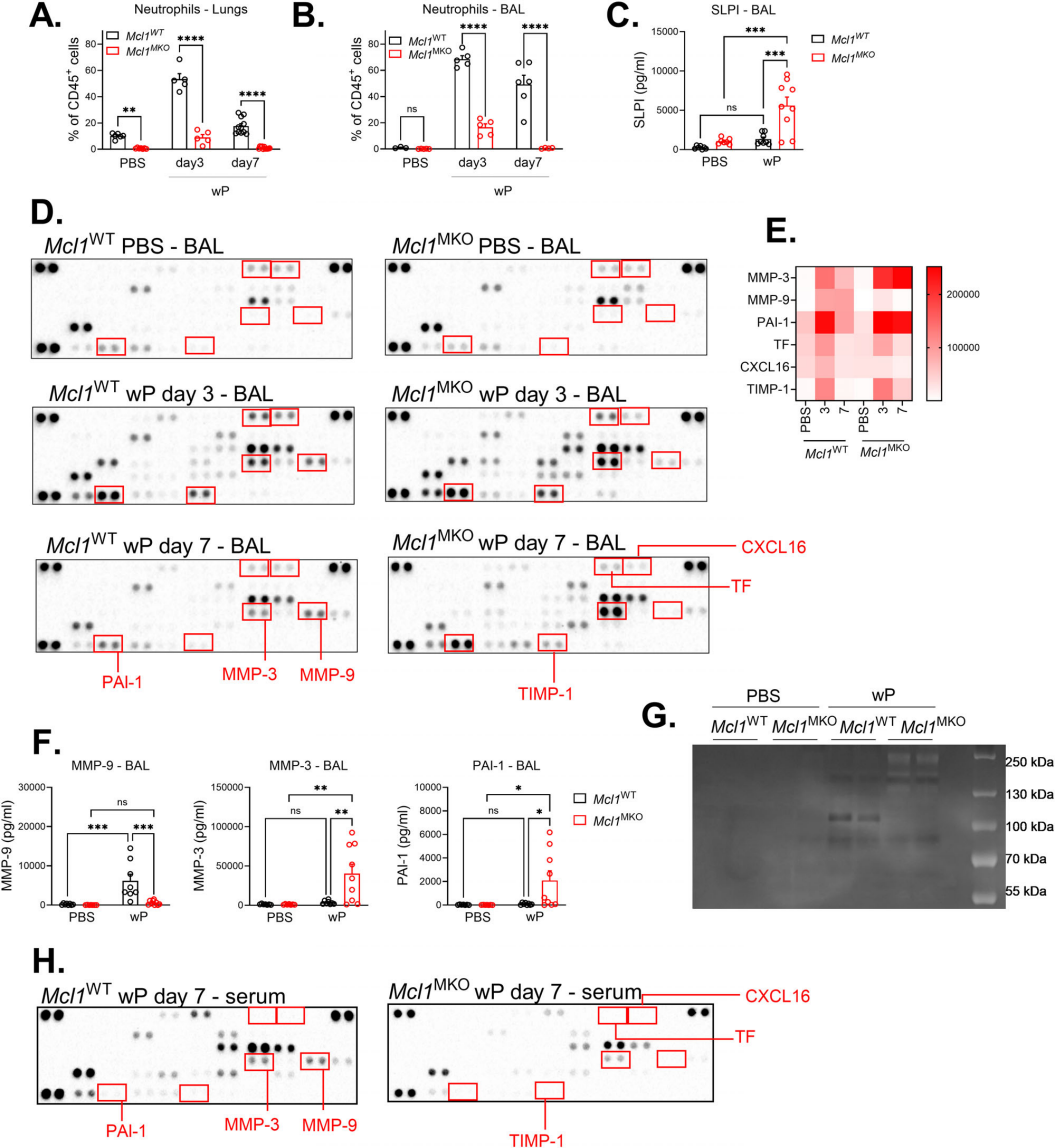
Neutrophils regulate early response to wP vaccine in the lungs. (A–H) *Mcl1* ^WT^ and *Mcl1* ^MKO^ mice were administered the wP vaccine intranasally. After 3 and 7 days, lungs, BAL cells and fluid were isolated. Neutrophil frequency in lungs (A) and BAL (B) was analysed by flow cytometry. (C) Analysis of SLPI level in BAL at day 7. (D) Proteome array analysis of pooled BAL fluids from 4 mice in each group. (E) Heatmap of densitometric analysis of membranes from (D). (F) Levels of MMP‐9, MMP‐3 and PAI‐1 were analysed by ELISA. (G) Representative zymography of BAL fluid samples from day 7. Each lane represents one mouse. (H) Proteome array analysis of pooled serum isolated from 4 mice per group. (A, B) Each point represents one mouse. For day 3, data represent 5 mice per experimental group from one experiment. For day 7, data represent 5–11 mice per experimental group from at least one independent experiment. PBS groups were pooled from day 3 and day 7 and represent 3–6 mice per experimental group from at least one independent experiment. Error bars show means ± SEM. **p < 0.01, ****p < 0.0001 by two‐way ANOVA, Tukey post hoc test. (C, F) Each point represents one mouse. Data represent 7–9 mice per experimental group pooled from three independent experiments. Error bars show means ± SEM. *p < 0.05, **p < 0.01, ***p < 0.001 by two‐way ANOVA, Tukey post hoc test. (D, H) Data represent one measurement per membrane from samples pooled from 4 mice.

We showed earlier that neutrophils are important producers of SLPI (Figure 1). To investigate the synergy between neutrophils and SLPI and their respective contribution to the release of MMP‐9 and other tissue mediators, we used neutrophil‐deficient mice [15]. Mice with neutrophil deficiency (*Mcl1* ^MKO^) and their control littermates (*Mcl1* ^WT^) were intranasally vaccinated with the PBS or wP vaccine for 3 or 7 days. As expected, neutrophil frequency in the lungs and BAL of *Mcl1* ^MKO^ mice was severely reduced (Figure 4A,B). Next, it was assessed whether neutrophil deficiency affects local mediators in BAL after wP vaccination. Interestingly, SLPI levels were significantly elevated in *Mcl1* ^MKO^ mice compared with controls (Figure 4C). Proteome array analysis of pooled BAL fluid collected at 3 and 7 days post‐vaccination revealed that neutrophil deficiency resulted in a marked decrease in MMP‐9, but increased levels of MMP‐3 and PAI‐1 in vaccinated mice (Figure 4D,E). Proteome findings were further validated by ELISA (Figure 4F). Furthermore, MMP‐9 activity, assessed by zymography, was detected exclusively in vaccinated *Mcl1* ^WT^ mice (Figure 4G). To confirm that the lack of neutrophils influences only the local response to i.n. vaccine, pooled serum samples were also analysed. Although proteome analysis did not show upregulation of MMP‐3 or PAI‐1 in serum of wP vaccinated *Mcl1* ^MKO^ mice, MMP‐9 was not detected (Figure 4H). These findings suggest two independent regulatory axes: neutrophil‐dependent production of specific mediators (e.g., MMP‐9) and a distinct, non‐neutrophil SLPI‐driven pathway. The pronounced increase in SLPI in neutrophil‐deficient mice supports its autonomous role in shaping the landscape of respiratory tissue mediators, including MMP‐3 and PAI‐1.

### 3.4 Exploratory Analysis of the Cellular Sources of Identified Mediators

**FIGURE 5:**
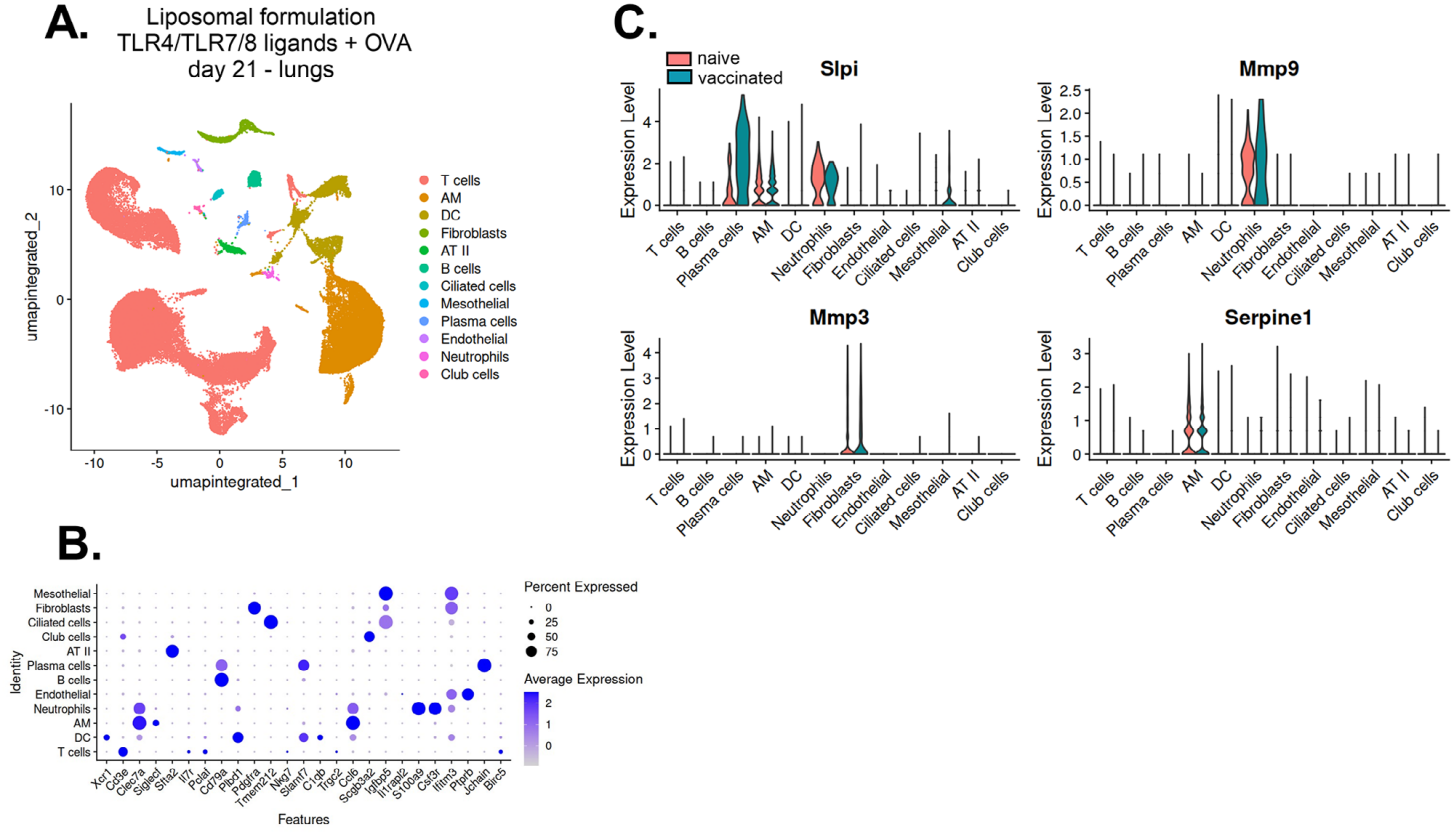
Cellular sources of various mediators in vaccinated mice. scRNA‐seq analysis of a publicly available dataset on lung cells isolated from control mice or mice administered with intranasal liposomal formulation with TLR4 and TLR7/8 ligands with OVA for 21 days. (A) UMAP of lung cells. (B) Dot plot of 12 clusters from lung cells. (C) Violin plots with expression levels of *Slpi*, *Mmp9*, *Mmp3*, and *Serpine1* transcripts in 12 clusters divided into control (red) and vaccinated (green) groups from lung cells.

To explore possible cellular sources of vaccine‐associated mediators, we analysed a publicly available single‐cell RNA sequencing (scRNA‐seq) dataset of lungs from naïve and vaccinated mice [17]. As there are no available datasets on lung cells from the intranasal wP vaccine, we selected the dataset that analysed cells from the lungs of mice that received intranasal liposomal formulation with TLR4 and TLR7/8 ligands with OVA for 21 days. Single lung cells were first clustered into immune populations (T cells, B cells, plasma cells, alveolar macrophages, dendritic cells, and neutrophils) and non‐immune populations (fibroblasts, endothelial cells, ciliated cells, mesothelial cells, alveolar type II cells, and club cells) (Figure 5A,B). Each cell type was then examined for expression of selected mediators in both naïve and OVA‐adjuvant‐vaccinated mice. *Slpi* transcripts were predominantly detected in neutrophils, alveolar macrophages, and plasma cells. Following vaccination, *Slpi* expression remained comparable in neutrophils and alveolar macrophages, whereas plasma cells exhibited upregulation of *Slpi* (Figure 5C). Next, genes of mediators that are upregulated by intranasal wP vaccination were analysed: *Mmp9* was restricted to neutrophils, *Mmp3* to fibroblasts, and *Serpine1* (encoding PAI‐1) to alveolar macrophages (Figure 5C). These results suggest that various lung cell populations might contribute to the release of specific soluble mediators after intranasal vaccination. However, independent studies are needed to elucidate the coordinated cellular response following wP vaccination.

### 3.5 Neutrophils Inhibit Local IgA Release Following Intranasal Vaccination

**FIGURE 6:**
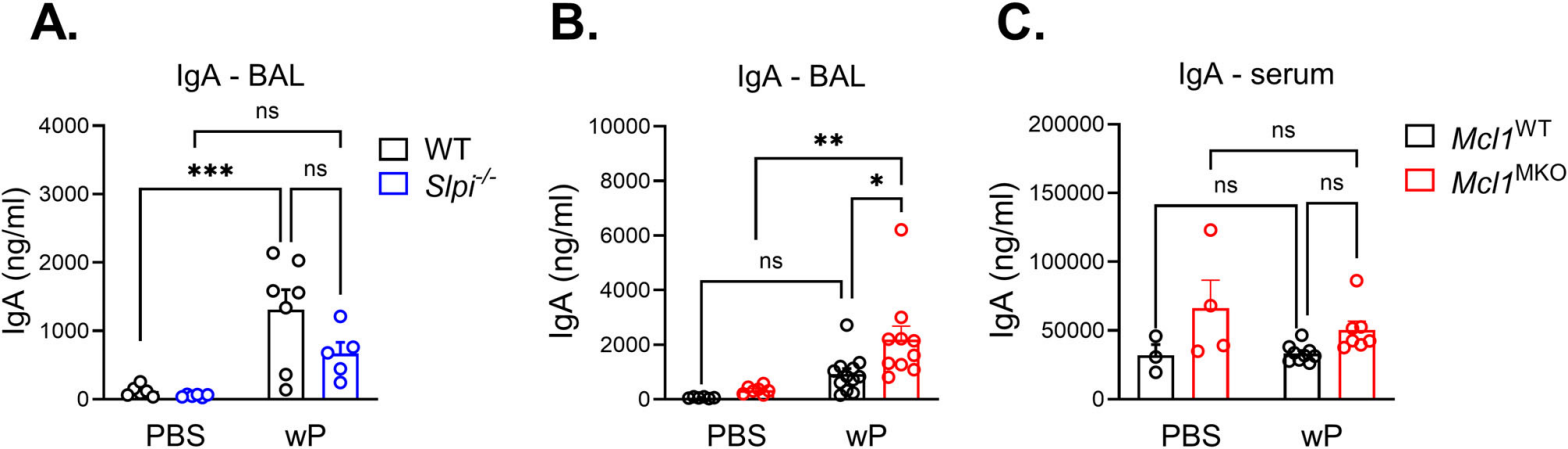
Total IgA secretion in wP vaccinated mice is regulated by SLPI and neutrophils. Mice were administered PBS as a control or wP intranasally and after 7 days BAL and serum were collected. (A) Analysis of BAL of WT and *Slpi* ^−/−^ mice for total IgA by ELISA. (B) Analysis of BAL from *Mcl1* ^WT^ and *Mcl1* ^MKO^ mice for total IgA by ELISA. (C) Analysis of serum of *Mcl1* ^WT^ and *Mcl1* ^MKO^ mice for total IgA by ELISA. (A–C) Each point represents one mouse. Data represent 3–12 mice per experimental group pooled from at least two independent experiments. Error bars show means ± SEM. *p < 0.05, ** *p* < 0.01, *** *p* < 0.001 by two‐way ANOVA, Tukey post hoc test.

Next, we examined whether SLPI or neutrophils influence early IgA secretion in the lungs after vaccination. Mice were immunised intranasally with PBS or wP, and on day 7 total IgA levels were measured in BAL fluid. As expected, vaccination with wP induced local IgA release. However, SLPI‐deficient mice tended to have reduced IgA levels after vaccination (Figure 6A), whereas neutrophil‐deficient mice showed a significant increase in IgA (Figure 6B). However, the increase in total IgA has not translated into antigen‐specific, anti‐heat‐killed *Bordetella pertussis* (HKBp) IgA antibodies. Their levels were very low in BAL, which can be due to the early time point of analysis; the responses were not yet detectable (Figure S2). In contrast to BAL, serum total IgA levels did not increase in neutrophil‐deficient mice following the vaccination (Figure 6C), which supports the local mucosal rather than systemic effect of the absence of neutrophils.

Collectively, these findings indicate that the balance between cellular contributors and soluble mediators, including neutrophils and SLPI, regulates local mediators and suggest that they might impact IgA secretion in the respiratory tract following intranasal vaccination.

## 4 Discussion

In recent years, there has been a major interest in developing vaccines that would target mucosal immunity to protect sites of pathogen entry and prevent both asymptomatic and symptomatic disease. Accordingly, the number of intranasal vaccines available in the clinic and in clinical trials is growing. However, to design an effective vaccine, there is a requirement to understand protective immunity acquired during natural infection or with available vaccines [21]. For instance, in the case of pertussis, bacterial infection and wP vaccine drive Th1/Th17 cellular responses in mice [22]. In contrast, aP vaccines induce Th2 responses and humoral responses which do not prevent pertussis colonisation and transmission [22, 23]. Furthermore, in recent years it has been proposed that contracting a pertussis infection may result in neurological complications leading to neuroinflammation and neurodevelopmental defects potentially due to local secretion of Bp toxin [24]. It was hypothesised that Bp is an important human neuropathogen and neuropathology may be initiated by nasopharyngeal subclinical Bp colonisation [25, 26]. Thus, intranasal immunisation may provide superior protection of the upper respiratory tract against Bp colonisation, protection of the olfactory surface and subsequently from the neuropathology induced by Bp colonisation.

Herein, we aimed to elucidate the early regulation and remodelling of the mucosal environment following intranasal administration of the wP vaccine. This approach mimics bacterial infection without the harmful effects of toxins produced by live Bp and may inform the design of future vaccines. Importantly, prior studies that have attempted to characterise the early immune response to intranasal vaccination have largely focused on the release of chemokines and cytokines [17, 27, 28]. Here, we extend this framework by uncovering previously uncharacterised tissue‐derived signals and immune regulators that govern these responses. We show that intranasal vaccination induces release of SLPI, MMP‐9, MMP‐3 and PAI‐1 into the bronchoalveolar space (Figure 1E, 2B). As SLPI and PAI‐1 are protease inhibitors [29, 30] and MMP‐3 and MMP‐9 are extracellular matrix enzymes [31], the role of these mediators might relate to tissue remodelling and vasculature changes following vaccination. Importantly, we show that these mediators are upregulated on day 3 post‐vaccination and they decrease at day 7 (Figures 2B and 4D). This suggests that these are transient effects in WT mice that can have an impact on immunity but do not cause any prolonged changes within the lungs.

Previously, SLPI has been shown to influence neutrophil functionality in the context of psoriasis, including the formation of neutrophil extracellular traps [32] as well as their migratory potential [33]. We did not observe a strong impact of SLPI on neutrophil recruitment to the respiratory tissues following intranasal immunisation within the studied time frame; however, secretion of neutrophil elastase was significantly elevated in SLPI‐deficient mice, suggesting a protective and regulatory role of SLPI in tissue damage and chronic inflammation (Figure 1J). Furthermore, our results demonstrate that SLPI plays a significant role in shaping local responses in the respiratory tract.

Neutrophils are cells whose functions are very well characterised in the context of the innate immune response and their role in protection against infections [34]. However, the influence of neutrophils on the response to vaccination has only recently begun to be investigated, and it remains unclear which of their specific functions affect long‐term immunity [35, 36, 37]. Our findings indicate that both SLPI and neutrophils are critical players in controlling the release of MMP‐3 and PAI‐1, as their lack induced local accumulation of these mediators (Figures 2 and 4). On the contrary, the release of MMP‐9 and IgA is regulated by SLPI and neutrophils with opposing effects (Figures 2, 4, and 6).

Paradoxically, although neutrophils are a major source of SLPI within immune cells, their absence is associated with an increase in local concentrations of this protease inhibitor (Figure 4C). Several mechanisms may explain the increase in SLPI accumulation. Airway epithelial cells and alveolar macrophages are important local sources of SLPI in the respiratory tract [19, 38]. In the absence of neutrophils, these cells may compensate by increasing SLPI production to maintain tissue homeostasis. Furthermore, the local response to vaccination in *Mcl1* ^MKO^ mice, which differ from *Mcl1* ^WT^, may involve factors that further enhance the expression of SLPI in the lungs. Alternatively, neutrophils release proteases, including neutrophil elastase and proteinase 3, both of which have been shown to degrade SLPI [39]. Therefore, the absence of neutrophils may reduce SLPI degradation, leading to its increased accumulation in the lungs.

It remains to be further investigated what the cellular sources of SLPI, MMP‐3, MMP‐9, PAI‐1 and also other mediators that were not tested in detail in our study are. We show from the scRNA‐seq dataset from lung cells of untreated and OVA plus adjuvant‐vaccinated mice that SLPI is expressed by various cells. SLPI expression has previously been reported in neutrophils, eosinophils and macrophages [19, 40, 41], but it remains to be validated whether plasma cells and mesothelial cells also express SLPI (Figure 5C). We also assessed the expression pattern of SLPI‐dependent mediators. As SLPI and PAI‐1 are both expressed by alveolar macrophages, it is possible that a lack of SLPI specifically in alveolar macrophages might lead to higher release of PAI‐1 into the bronchoalveolar space (Figure 2C). In contrast, MMP‐3 is expressed by fibroblasts, but this subset does not express SLPI at detectable levels. Therefore, the regulation of MMP‐3 by SLPI might be indirect. Furthermore, we show that neutrophils express SLPI and are the main source of MMP‐9 release into the bronchoalveolar space during vaccination (Figures 1 and 3). It has been previously described that there is an increase in MMP‐9 release in the absence of SLPI in the context of lung injury [20] and by tissue‐resident peritoneal macrophages [19]. Furthermore, neutrophils have been shown to upregulate MMP‐9 after Bp infection [42], suggesting that neutrophil activation is uniform for both natural infection and vaccination. Finally, we also assessed early adaptive immunity in the context of local total IgA levels. IgA was increased following vaccination in WT mice, and it was observed that IgA levels correlate with SLPI release. In SLPI‐deficient mice, IgA production had the tendency to decrease, while in neutrophil‐deficient mice, which show upregulation of SLPI, IgA production was significantly increased.

Recent studies have reported a positive correlation between SLPI expression and the abundance of IL‐17‐producing cells in the intestinal mucosa of patients with inflammatory bowel disease [43]. In turn, IL‐17 has been shown to promote mucosal IgA responses by enhancing epithelial expression of the polymeric immunoglobulin receptor (pIgR), which mediates the transport of secretory IgA, and by supporting the development of antigen‐specific IgA responses at mucosal sites [10, 44, 45, 46]. Although IL‐17^+^ cells were not investigated in the present study, it is possible that elevated SLPI levels in *Mcl1* ^MKO^ mice are associated with an altered IL‐17 response that contributes to the increased total IgA observed in the airways. On the contrary, neutrophils have been reported to negatively regulate the induction of mucosal IgA responses after immunisation [47], although the mechanisms underlying this regulatory effect remain poorly understood. Overall, our findings support the role of neutrophils and SLPI in shaping mucosal IgA responses, but the precise interplay between SLPI, neutrophils, IL‐17, and adaptive immunity following vaccination, including the wP vaccine, requires further investigation.

SLPI also exhibits anti‐apoptotic properties across various cell types [48, 49], whereas NET formation is associated with increased apoptosis of B lymphocytes and a reduced number of IgA‐producing plasma cells [50]. Thus, SLPI might also contribute to plasma cell maintenance and, consequently, to enhanced IgA production, whereas neutrophils suppress secretion of IgA after intranasal delivery of wP vaccine.

Many vaccines delivered intramuscularly fail to generate respiratory mucosal protection. As the olfactory mucosa is separated from circulating antibodies and immune cells by the blood–endothelial barrier, there is potentially a requirement for tissue remodelling to enable the development of effective immunity. Our data show that specific mediators are released early following intranasal vaccination, and they might facilitate transient tissue remodelling in the context of intranasal vaccination. At the same time, they have been associated with tissue fibrosis, asthma, and thrombosis when chronically released [51, 52, 53]. Thus, the issue of balancing the strength of inflammation with the long‐term immune response induced by vaccines, one that provides safe protection against pathogens, remains to be determined.

### 4.1 Data Limitations and Future Perspectives

In this study, we investigated the early mucosal immune response to intranasal whole‐cell pertussis vaccination, focusing on airway SLPI, neutrophils, and soluble mediators associated with tissue remodelling and coagulation. However, several limitations should be acknowledged.

First, although the proteome profiler identified multiple differentially regulated mediators, only a subset was validated in independent experiments. Therefore, the remaining candidates require confirmation in future studies. Second, the cellular source of SLPI released into the bronchoalveolar space after vaccination with wP in neutrophil‐deficient mice was not determined. Third, although we analysed an existing lung scRNA‐seq dataset to provide additional mechanistic insight, these findings cannot be directly extrapolated to the wP vaccine because the dataset represents a different vaccine formulation and time point. However, it provides a useful starting point for future investigations. Finally, our study focused on the early response to intranasal vaccination and therefore assessed only total IgA in the bronchoalveolar space. Antigen‐specific IgA levels were too low to allow conclusions regarding the role of SLPI or neutrophils in the development of protective immunity. Studies incorporating at least two doses of vaccine and longer follow‐up periods will be required to comprehensively evaluate adaptive immune responses to intranasal wP vaccination, including antigen‐specific antibody production and T cell responses.

Despite these limitations, our findings identify early airway mediators induced by intranasal wP vaccination that may represent candidate biomarkers for the development and optimisation of future mucosal vaccines.

Given the high expression of SLPI in nasopharyngeal tissues, its early assessment may help predict optimal vaccine formulations, particularly with respect to adjuvant and antigen selection. Overall, our findings can also inform controlled human infection models (CHIMs) to study the immune response in a respiratory mucosa after Bp infection in humans and to test new intranasal vaccines against Bp and potentially other pathogens.

## Author Contributions

**Oktawia Osiecka**: investigation, formal analysis, writing – reviewing and editing. **Mariia Tyshchenko**: investigation, formal analysis, writing – reviewing and editing. **Ivan Sinkevich, Natalia Pocałuń**: investigation. **Joanna Cichy**: resources, writing – reviewing and editing. **Jakub M. Kwiecinski**: methodology, resources, writing – reviewing and editing. **Ewa Oleszycka**: investigation, software, formal analysis, visualisation, funding acquisition, resources, writing – original draft, reviewing and editing. **Mieszko M. Wilk**: conceptualisation, funding acquisition, resources, methodology, investigation, formal analysis, visualisation, project administration, supervision, writing – original draft, reviewing and editing.

## Conflicts of Interest

Mieszko M. Wilk reports a relationship with IBSS BIOMED S.A., Poland that includes consulting or advisory, employment, and paid expert testimony. The remaining authors declare no conflicts of interest.

## Supporting information




**Supporting File**: eji70282‐sup‐0001‐SuppMat.pdf.

## Data Availability

Raw data supporting the findings of this study can be found in the public repository: https://doi.org/10.57903/UJ/XF7RFZ.
