# Supplementary material for: Loss of Neutrophils and SLPI Reshapes Early Mucosal Inflammatory Responses During Intranasal Vaccination
Source: Eur J Immunol. 2026 Sep 16;56(9):e70282. doi: 10.1002/eji.70282 (PMC13579227; doi:10.1002/eji.70282)
Supplement: Supplementary file 1 [file EJI-56-e70282-s001.pdf]

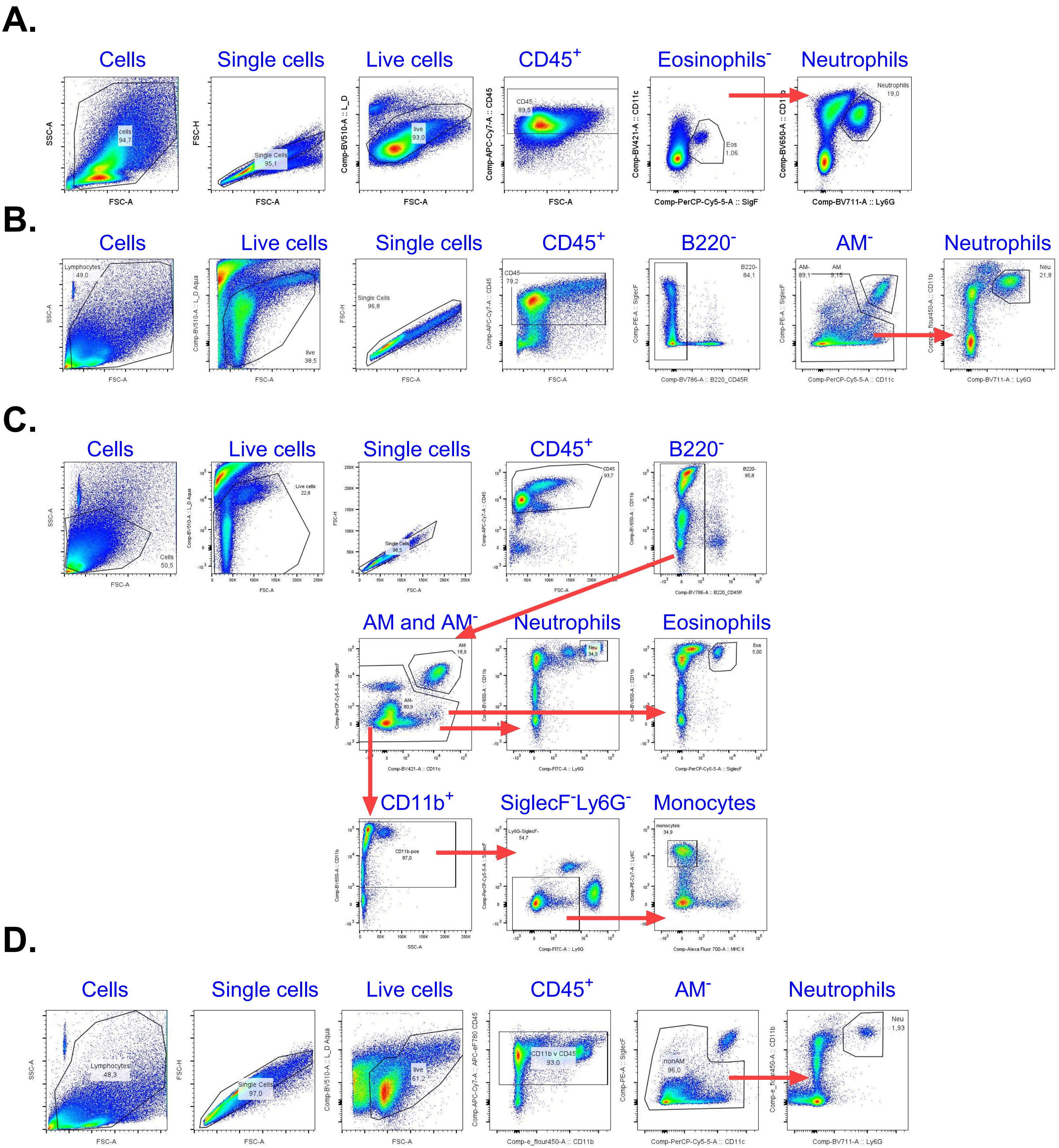

Supplementary Figure 1. Gating strategy for flow cytometry analysis. (A) Neutrophil population gating in PEC - Figure 1. Neutrophils were gated as live, single CD45^{+}SiglecF^{-}CD11b^{+}Ly6G^{+} cells. (B) Neutrophil population gating in lung and BAL - Figure 1 and 4. Neutrophils were gated as live, single CD45^{+}B220^{-}CD11c^{-}SiglecF^{-}CD11b^{+}Ly6G^{+} cells. (C) Alveolar macrophages (AM), monocytes, neutrophils and eosinophils - Figure 1. (D) Neutrophil population gating in lung - Figure 3. Neutrophils were gated as live, single CD45^{+}CD11c^{-}SiglecF^{-}CD11b^{+}Ly6G^{+}cells.

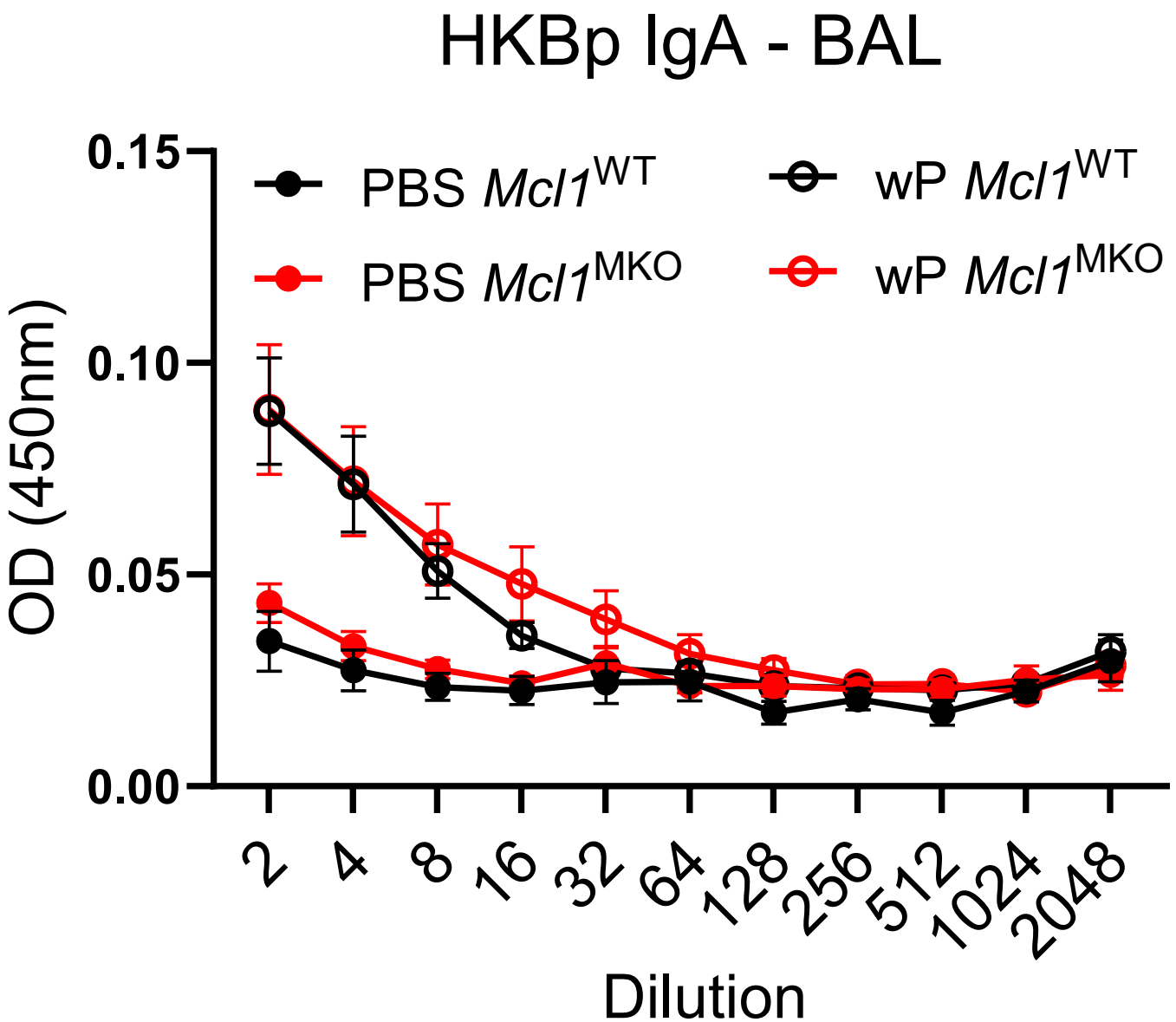

Supplementary Figure 2. HKBp-specifc IgA antibodies at day 7 following vaccination. *Mcl1*^{WT} and *Mcl1*^{MKO} mice received one dose of PBS or wP vcacine intranasaly. Analysis of serially diluted BAL. Data represent 6 to 12 mice per experimental group pooled from three independent experiments. Error bars show means ± SEM.
